# Conflict components of the Stroop effect and their “control”

**DOI:** 10.3389/fpsyg.2014.00463

**Published:** 2014-05-20

**Authors:** Yulia Levin, Joseph Tzelgov

**Affiliations:** ^1^Department of Psychology, Ben-Gurion University of the NegevBeer Sheva, Israel; ^2^Department of Brain and Cognitive Sciences, Zlotowski Center for Neuroscience, Ben-Gurion University of the NegevBeer Sheva, Israel

**Keywords:** Stroop effect, task conflict, informational conflict, conflict adaptation, cognitive control, conflict monitoring

## A genuine marker of the automaticity of reading in the stroop task

For the past four decades or so, an idea contrasting early definitions of automaticity and claiming that automatic processes can be controlled, has dominated the literature (Logan, 1980, 1985; Norman and Shallice, 1986; Tzelgov et al., 1990). The interference effect found in the Stroop task is usually considered to be a marker of automaticity of reading, while the modulation of its magnitude is referred to as a marker of control. In the present article, we emphasize the frequently overlooked notion that what we refer to as a marker of automaticity has in fact multiple origins. MacLeod and MacDonald (2000) and Goldfarb and Henik (2007) suggested that two types of conflict—the task and informational conflicts—contribute to Stroop interference. The *informational conflict* (henceforth IC) represents competition between two color concepts: one that is activated through color naming and the second that is activated by the reading process (e.g., the concepts red and blue respectively, when the stimulus is the word BLUE written in red ink). However, according to the same authors, some amount of interference is obtained even with color-unrelated words, since all words automatically activate the irrelevant reading task, setting in motion the competition between two possible tasks (henceforth *task conflict*; TC) (see Kalanthroff et al., 2013a; Entel et al., submitted, for behavioral evidence, and Bench et al., 1993; Carter et al., 1995, for neuroimaging evidence of the TC). Even non-word stimuli containing lexical information (e.g., letter strings) can interfere because they are readable (Klein, 1964; Sharma and McKenna, 1998). The more word-like the stimulus, the more interference it produces (Monsell et al., 2001). Thus, as evident from this distinction, the genuine marker of automaticity is the TC whereas the IC amplifies the interference from the irrelevant task. That is, in order to argue that the automatic reading process can be controlled one should actually show that what is controlled is the TC.

## List-wide proportion congruent effect and the conflict adaptation account

**Figure 1:**
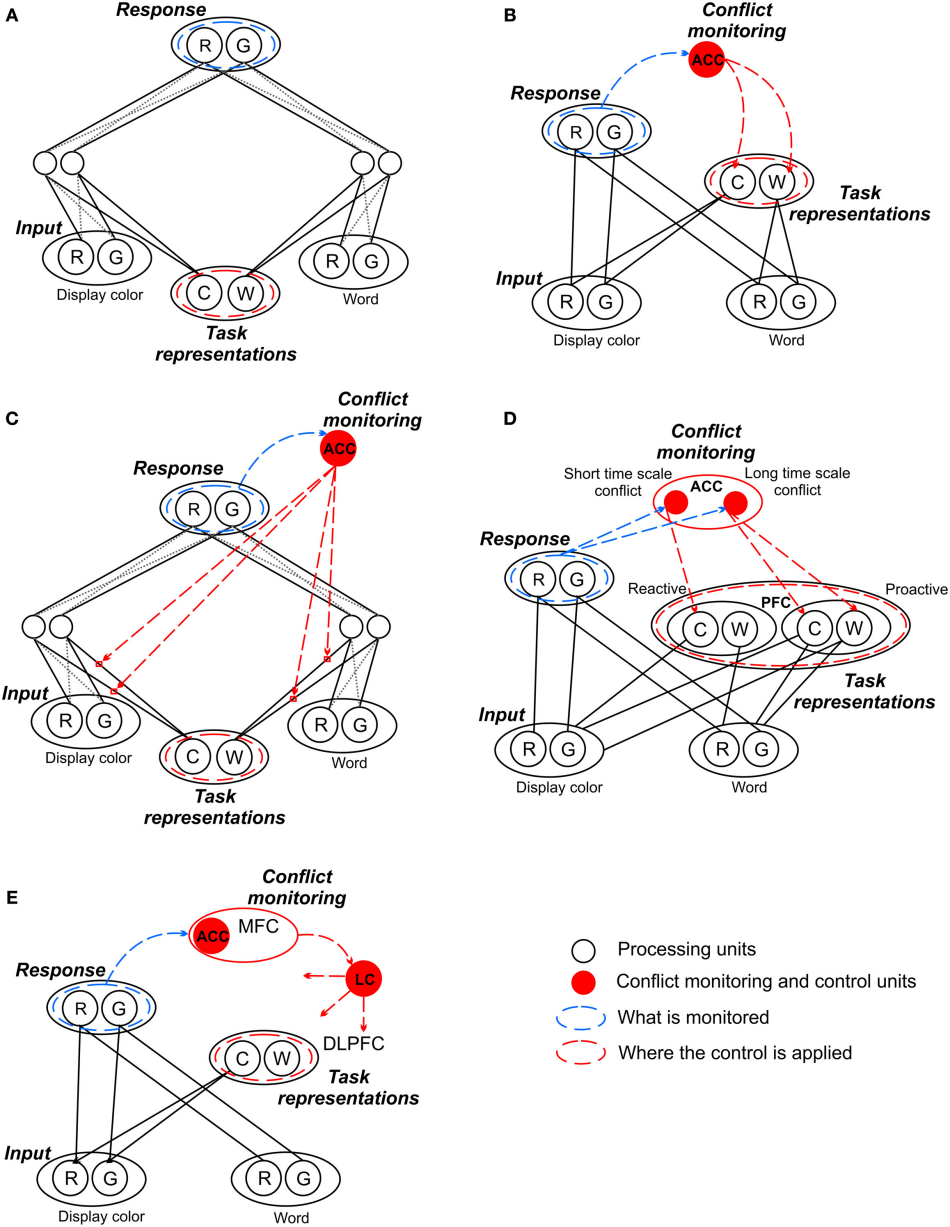
**The proposed architectures within the conflict-monitoring framework. (A)** All models share the same core architecture introduced by Cohen et al. (1990) in their explanation of Stroop effect performance. This includes the definition of conflict as *response competition*, implying an aggregated contribution of task conflict and informational conflict. The assumption that conflict is controlled solely by adjustment of *task representation* weights implies that only the task conflict can be directly controlled. **(B)** Botvinick et al.'s (2001, 2004) model added a conflict-monitoring unit thereby generating a control loop for adjusting the task representation weights, while **(C)** Blais et al. (2007) proposed that task weights can be adjusted differentially for specific items. **(D)** De Pisapia and Braver's (2006) architecture captures the distinction between reactive and proactive control. **(E)** Verguts and Notebaert's (2008, 2009) model suggests control is modulated through conflict-based Hebbian learning. Note the models are depicted in a very schematic way, with no reference to the nature and direction of the existed connections, their specific weights, etc. The detailed information can be found in the original articles (see references). R, red; G, green; C, color; W, word; ACC, anterior cingulate cortex; LC, locus coeruleus; MFC, medial frontal cortex; PFC, prefrontal cortex; DLPFC, dorsolateral prefrontal cortex.

The more frequent the incongruent trials are in an experiment, the smaller the Stroop effect (Logan and Zbrodoff, 1979; Logan, 1980; Tzelgov et al., 1992). This is known as the *list-wide proportion congruent* effect because the proportions are manipulated at the list level. The list-wide proportion congruent effect is considered to be a marker of control since it demonstrates the modulation of the magnitude of the Stroop effect, and as such, is interpreted in terms of conflict adaptation. According to the conflict-monitoring framework (Botvinick et al., 2001; De Pisapia and Braver, 2006; Braver, 2012), an increased proportion of incongruent trials results in higher conflict at the response level, which triggers the control system. The control process includes two stages: conflict detection and control exertion. Referring to our previous discussion, in order to claim the automatic reading process can be controlled, the TC should be the target of both stages of the control process. However, according to our analysis, this is not the case. In fact, the TC is only a target of the control exertion stage. According to the models within the conflict-monitoring framework, conflict reduction is achieved through adjusting the weights of the two tasks, thereby minimizing the TC. However, the conflict detection stage is centered on response competition, which requires the TC to be amplified by the IC. When there is no IC, that is, no competing color-concept activation by reading, no competing color-response can be activated. Focusing on response competition (and thereby on IC) by Botvinick et al. and later models (De Pisapia and Braver, 2006; Blais et al., 2007; see also Verguts and Notebaert, 2008, for a model integrating cognitive control and reinforcement learning) leads to the conclusion that the detection of conflict, and therefore triggering of the entire control process, requires the IC being present (see Kalanthroff et al., 2013b, for evidence inconsistent with this assumption). There is no “path” in these architectures allowing TC to be monitored without the presence of the IC (Figure 1). That is, the theory behind these architectures in their current state does not allow an unequivocal claim that reading, as an automatic process, can be controlled.

## Item-specific proportion congruent effect: Conflict adaptation vs. learning account

The conflict adaptation account has been challenged by manipulating the proportions of incongruent trials at the item level, revealing an *item-specific proportion congruent* effect (Jacoby et al., 1999, 2003). In the item-specific paradigm, list-wide proportion congruence is held at 50%, and specific words are paired in most of the trials with a specific color, creating mostly congruent (i.e., not associated with conflict) or mostly incongruent (i.e., strongly associated with conflict) stimuli. The item-specific proportion congruent effect refers to a smaller interference for mostly incongruent items than for mostly congruent items.

In order to determine whether a word causes conflict, the word should be read, which contradicts the main assumption of the models in the conflict-monitoring literature, including those specifically adapted to explain the item-specific findings (Blais et al., 2007), that control operates proactively to prevent the initiation of the reading process. Consequently, it has been proposed (Bugg et al., 2008, 2011), and supported by empirical data (Bugg and Hutchison, 2012; Abrahamse et al., 2013), that item-specific control may be based on reactive control. This idea, however, is inconsistent with the assumption that reading, as an automatic process, is ballistic (Bargh, 1989), that is, difficult to stop once started. Stopping a ballistic reading process seems especially unlikely given that it is completed extremely quickly (Sereno et al., 1998; Cohen et al., 2000; Pulvermuller et al., 2001). More important, as the previous section illustrates, the conflict adaptation account can only explain the pattern obtained for incongruent (i.e., producing IC) items in mostly incongruent and mostly congruent conditions. However, manipulating the proportions at the item level seems also to affect the congruent (i.e., producing no IC) items, as evidenced (in our view) by the results of Jacoby et al. (2003). In that study, a 50/50 condition in which the number of presentations of words in each color was equal for congruent or incongruent stimuli was included in addition to the mostly congruent and mostly incongruent conditions. The analysis carried out by the authors showed that the larger the proportion of incongruent items was (from mostly congruent to 50/50 to mostly incongruent), the lesser the Stroop effect obtained. However, the 50/50 condition can be defined not just as a condition including more incongruent items than the mostly congruent condition, but also as a *neutral* condition where the conflict cannot be predicted by reading. Jacoby et al.'s data reveal that in comparison to the “neutral” (50/50) condition, incongruent items in the mostly incongruent condition were 32 ms faster. Similarly, and surprisingly, congruent items in the mostly congruent condition also showed a 21 ms reaction time (RT) reduction. Identical information regarding the conflict is provided by reading congruent words in the 50/50 and mostly congruent conditions, and yet RT in the latter condition is faster. This pattern contradicts the conflict adaptation account since congruent items do not produce IC, which according to our analysis, is the basis for control modulation. Schmidt et al. (2007; Schmidt and Besner, 2008) proposed a *contingency learning* account to explain Jacoby et al.'s (2003) finding without assuming conflict adaptation. It postulates that pairing a word with a specific color creates an association between that word and a specific response. The mechanism of contingency learning functions by lowering the threshold of the most frequently encountered response to the word, and does not lower the thresholds of other possible responses. Since according to the contingency learning account it does not matter if the word is paired mostly with congruent or incongruent colors, the facilitative effect of learning predicted by the contingency learning account is consistent with the results of Jacoby et al. (2003).

To prove the independence of the contingency learning mechanism of conflict, Schmidt and Besner (2008) demonstrated that the effects of contingency learning and congruency (i.e., IC) are additive by reanalyzing Jacoby et al.'s (2003) data. This evidence, however, is somewhat problematic because the rearrangement of the cells in the design manipulating proportion congruency still has the (congruency) confound, and the effect of contingency learning cannot be validly evaluated in such an analysis. In order to test directly whether contingency learning depends on the presence of conflict, Schmidt et al. (2007) (also Schmidt and Besner, 2008) conducted an experiment in which they eliminated IC by using neutral (i.e., color-unrelated) words only as stimuli in a color naming task. Their results demonstrated that the contingency learning effect does not require a stimulus to be a color-related (i.e., conflicting) word, supporting the idea that contingency learning is independent of the presence of IC. However, as suggested by MacLeod and MacDonald (2000), even neutral words are conflicting with respect to TC. Thus, the contingency learning effect might be independent of IC, but not of TC. Although such dependency would not weaken the ability of the contingency learning to explain the item-specific proportion congruent effect, it would suggest that this account might actually represent another control-like adaptive mechanism activated by (task) conflict. In fact, such evidence would dissipate the core controversy (i.e., control vs. learning) around the interpretation of the conflict adaptation effect, by incorporating the contingency learning into the category of control mechanisms.

Another potential problem with the contingency learning account is that it assumes that the association learned refers to a particular response in the sense of the button that should be pushed, but not in the sense of the correct color. This claim, supported by the results of their Experiment 4, is explicitly stated by Schmidt et al. (2007): “… pairings of stimuli do not simply form semantic connections… but instead directly cause changes in our behavior …” It is also evident in the architecture of the proposed parallel episodic processing (PEP) model (Schmidt, 2013) where the response generation layer consists of representations of the buttons the responses are mapped to, but not of the response set colors. If so, then it posits the questions of what would happen, and how contingency learning would express itself when instead of pushing the keys on a keyboard, responses are made vocally. When the response requires naming the color, there is no other way contingency learning can proceed but through linking the word with a specific color-concept because the latter is necessary for making a verbal response. That is, with vocal responses, contingency learning is predicted to affect the informational and not the response level of representations. However, if the words already have a strong semantic association with the color concept (i.e., congruent condition) then the contribution of the contingency learning process should be minimal, if at all. Therefore, with respect to the current discussion, the congruency of the item, or in other words, informational conflict or its absence, in some situations, might matter even for the contingency learning process.

## Summary

We do not pretend that the distinction between task and information conflict can solve the ongoing argument regarding the mechanism behind the “flexibility” of the Stroop effect, as reflected by the proportion effect. We do believe that the awareness of the fact that only one of two components contributing to the Stroop effect is a genuine marker of the automaticity of reading, would undoubtedly help in further developing existing control models, and probably new ones, that would be able to answer the question regarding controllability of reading. Distinguishing between two types of conflict can also be valuable with respect to the “control vs. learning” debate. For now, the proposed learning mechanism (i.e., contingency learning), as an alternative explanation for some of the proportion congruent effects, has only been proven to be independent from the IC. However, as mentioned, the TC is what really matters. Hence, in order to be considered as an independently standing mechanism that is not part of the control system, the contingency learning should also be evident when no TC is produced by stimuli.

### Conflict of interest statement

The authors declare that the research was conducted in the absence of any commercial or financial relationships that could be construed as a potential conflict of interest.
